# AI-driven identification of nutrition-modulated biomarkers and drug targets for cardiovascular therapeutic mechanisms

**DOI:** 10.3389/fphar.2026.1793532

**Published:** 2026-03-09

**Authors:** Ye Luo, Yuhan Mou, Zhaoting Li, Bin Liao, Juyi Wan

**Affiliations:** 1 Department of Cardiovascular Surgery, The Affiliated Hospital, Southwest Medical University, Metabolic Vascular Diseases Key Laboratory of Sichuan Province, Key Laboratory of Cardiovascular Remodeling and Dysfunction, Luzhou, Sichuan, China; 2 Key Laboratory of Medical Electrophysiology, Ministry of Education and Medical Electrophysiological Key Laboratory of Sichuan Province, (Collaborative Innovation Center for Prevention of Cardiovascular Diseases), Institute of Cardiovascular Research, Southwest Medical University, Luzhou, Sichuan, China; 3 Department of Cardiothoracic Surgery, Luzhou Hospital of Traditional Chinese Medicine, Luzhou, Sichuan, China

**Keywords:** artificial intelligence, biomarkers, cardiovascular disease, drug targets, network pharmacolog, nutrition-modulated pathways, precision pharmacology

## Abstract

Cardiovascular diseases (CVD) remain the leading cause of disease burden and mortality worldwide. Despite significant progress in drug treatment, this situation indicates that persistent residual risks still exist even after all feasible risk control measures have been implemented. Nutrition is increasingly recognized as an important modulator of cardiovascular biology; however, its integration into pharmacological frameworks for biomarker discovery and drug target identification has remained limited, largely due to insufficient mechanistic resolution and analytical complexity. Recent progress in high-throughput multi-omics technologies has revealed that nutrients and nutrient-derived metabolites directly regulate key pathways involved in lipid metabolism, inflammation, and mitochondrial function, many of which overlap with established or emerging cardiovascular drug targets. In parallel, artificial intelligence (AI) has emerged as a powerful discovery engine capable of integrating high-dimensional nutritional, molecular, and clinical data to prioritize biomarkers and uncover therapeutically actionable targets. In this mini-review, unlike previous studies that focused on dietary patterns and behavioral recommendations, we have summarized the current evidence regarding the drugable pathways for nutritional regulation in cardiovascular diseases, and have particularly highlighted the strategies based on artificial intelligence - including machine learning, network pharmacology, and multi-omics integration - for identifying biomarkers and elucidating therapeutic mechanisms. We further discuss the translational implications of AI-enabled nutritional pharmacology for precision cardiovascular therapeutics. By reframing nutrition as a source of modifiable molecular signals rather than a lifestyle exposure, this review provides a mechanistic framework for harnessing AI to advance biomarker discovery and drug target identification in cardiovascular disease.

## 1 Introduction

Cardiovascular disease (CVD) remains the leading cause of mortality worldwide despite substantial advances in pharmacological therapies targeting lipid metabolism, thrombosis, and inflammation (Global Burden of Cardiovascular Diseases and Risks, 2025). Although lipid-lowering agents, antiplatelet drugs, and anti-inflammatory strategies have significantly improved clinical outcomes, a considerable proportion of patients continue to experience residual cardiovascular risk (Reijnders et al., 2023). This limitation highlights the need for novel therapeutic frameworks that move beyond single-target interventions and better reflect the molecular complexity of cardiovascular pathology (Lin et al., 2025; de Ruiter et al., 2025).

Nutrition has long been recognized as a major determinant of cardiovascular health; however, its role has traditionally been interpreted through epidemiological associations and lifestyle-based risk modification (Martínez-González et al., 2025). Such approaches, while valuable for prevention, provide limited mechanistic insight and have weak translational relevance for drug discovery (Stouras et al., 2022). Therefore, a simple nutritional intervention is unlikely to effectively address the issue of cardiovascular diseases. Increasing evidence suggests that nutrients and nutrient-derived metabolites exert direct regulatory effects on molecular pathways central to atherosclerosis, endothelial dysfunction, and myocardial injury, including lipid handling, inflammatory signaling, and cellular energy metabolism (Mallick and Duttaroy, 2022; Tonch-Cerbu et al., 2025). Importantly, many of these pathways overlap with established or emerging pharmacological targets, indicating that nutritional inputs can be conceptualized as endogenous modulators of druggable biological systems rather than nonspecific environmental factors (Stouras et al., 2022).

The rapid expansion of high-throughput omics technologies has further revealed the molecular intermediates linking nutrition to cardiovascular disease (Dong et al., 2023; Zhang and Schmidlin, 2024). Large-scale metabolomic and lipidomic studies have identified circulating metabolites and lipid species associated with cardiovascular risk and plaque instability, providing a rich source of candidate biomarkers with potential therapeutic relevance (Buergel et al., 2022; Gong et al., 2025; Harm et al., 2023). However, the high dimensionality and heterogeneity of these datasets pose substantial analytical challenges, limiting their direct application to pharmacological target discovery.

Artificial intelligence (AI) has emerged as a powerful tool to address these challenges by enabling systematic integration and interpretation of complex biological data (Chen et al., 2024; Nam et al., 2024). Machine learning and network-based approaches can identify non-linear relationships, prioritize nutrition-responsive biomarkers, and uncover key regulatory nodes within molecular networks that are amenable to pharmacological intervention (de Ruiter et al., 2025). Recent studies demonstrate that AI-driven multi-omics integration improves cardiovascular risk stratification and facilitates the identification of biologically meaningful targets that may not be detectable using conventional statistical methods.

**FIGURE 1:**
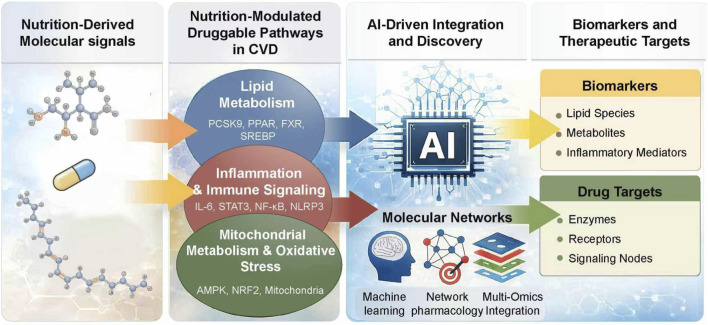
Al-enabled framework for identifying nutrition-modulated biomarkers and drug targets in cardiovascular disease.

By reframing nutrition as a source of modifiable molecular signals and leveraging AI as a discovery engine, a new paradigm is emerging in cardiovascular pharmacology. Previous studies have demonstrated interventions for cardiovascular diseases through healthy dietary patterns or exercise, but the certainty of these interventions is relatively low. Moreover, more research is needed to study the specific populations and the interactions between nutrients. Rather than focusing on dietary patterns or behavioral recommendations, this framework emphasizes the identification of nutrition-modulated biomarkers and druggable pathways with direct translational potential. In this mini-review, we summarize current evidence on nutrition-responsive molecular mechanisms in cardiovascular disease, highlight AI-driven strategies for biomarker and target identification, and discuss their implications for precision cardiovascular therapeutics (Figure 1).

## 2 Nutrition-modulated druggable pathways in cardiovascular disease

Nutrition influences cardiovascular disease (CVD) progression primarily through modulation of molecular pathways that govern lipid metabolism, inflammation, and cellular energy homeostasis (Tonch-Cerbu et al., 2025; Kong et al., 2022; Wculek et al., 2022). Importantly, many of these pathways (such as lipid metabolism, inflammatory response, mitochondrial dysfunction, etc.) are already established or emerging targets in cardiovascular pharmacology, underscoring the potential to reposition nutritional signals as endogenous modulators of druggable biological systems rather than nonspecific lifestyle factors (Ray et al., 2020).

### 2.1 Lipid metabolite drug targets

Lipid and cholesterol metabolism represents the most direct and pharmacologically tractable interface between nutrition and cardiovascular disease (Xia et al., 2021). Dietary lipids and cholesterol influence circulating lipoprotein profiles, hepatic lipid handling, and vascular lipid deposition, thereby shaping atherosclerotic plaque development and stability (Mateu-Fabregat et al., 2024). Central regulatory nodes in this process include proprotein convertase subtilisin/kexin type 9 (PCSK9), sterol regulatory element-binding proteins (SREBPs), peroxisome proliferator-activated receptors (PPARs), and the bile acid–activated nuclear receptor farnesoid X receptor (FXR) (Zo et al., 2025; Montaigne et al., 2021; Ding et al., 2024).

Nutritional status and nutrient-derived metabolites exert substantial control over these signaling axes (Tonch-Cerbu et al., 2025). For example, fatty acid composition modulates PPAR activity, while bile acids—whose synthesis and composition are influenced by dietary fat intake and gut microbiota—serve as endogenous ligands for FXR (Zhang et al., 2024). These pathways directly overlap with successful pharmacological strategies, including PCSK9 inhibitors and PPAR agonists, highlighting their druggability and clinical relevance (Das Pradhan et al., 2022). Recent multi-omics studies further demonstrate that specific lipid species and bile acid profiles, shaped in part by nutrition, correlate with plaque vulnerability and cardiovascular events, supporting their potential role as both biomarkers and therapeutic entry points (Gong et al., 2025; Chong Nguyen et al., 2021).

Rather than treating dietary lipids solely as risk modifiers, these findings position lipid-regulated signaling pathways as dynamic, nutrition-responsive targets that can be integrated into cardiovascular drug discovery and optimization strategies.

### 2.2 Inflammation and immunity drug targets

Chronic low-grade inflammation is a defining feature of atherosclerosis and myocardial injury, and its central role in CVD has been validated by the clinical success of anti-inflammatory therapies (Nidorf et al., 2020; Tardif et al., 2019). Nutritional factors modulate inflammatory signaling through effects on immune cell metabolism, cytokine production, and redox balance (Hou et al., 2023). Nutrient availability influences key inflammatory pathways, including IL-6/STAT3, nuclear factor-κB (NF-κB), and the NLRP3 inflammasome, which act as convergence points linking metabolism and immune activation (Ridker et al., 2021; Chen and Li, 2024).

Diet-derived metabolites, such as certain fatty acids (acetyl coenzyme A and ketone bodies, etc.) and amino acid derivatives (neurotransmitters and signaling molecules, etc.), can alter macrophage polarization and endothelial inflammatory responses, thereby shaping plaque development and progression” (Eshghjoo et al., 2022). Importantly, these inflammation-associated pathways are pharmacologically actionable, as evidenced by targeted anti-cytokine strategies and inflammasome inhibitors (Wohlford et al., 2020). The overlap between nutrition-regulated inflammatory signaling and established drug targets suggests that nutritional modulation may refine patient stratification and therapeutic responsiveness rather than simply reduce baseline risk.

This perspective supports a shift from viewing nutrition as an external modifier of inflammation toward recognizing it as an intrinsic regulator of immune-metabolic drug targets in cardiovascular disease.

### 2.3 Oxidative stress and mitochondrial dysfunction drug targets

Oxidative stress and mitochondrial dysfunction contribute to endothelial injury, vascular remodeling, and myocardial damage across multiple stages of cardiovascular disease (Kopych et al., 2025; Poznyak et al., 2020). Nutrient availability and composition influence mitochondrial energetics and redox homeostasis through signaling regulators such as AMP-activated protein kinase (AMPK) and nuclear factor erythroid 2–related factor 2 (NRF2) (Qiu et al., 2023; Chen, 2021). These pathways integrate cellular energy status with antioxidant defenses and metabolic adaptation.

Rather than supporting nonspecific antioxidant supplementation, contemporary evidence emphasizes modulation of upstream metabolic signaling as a more effective therapeutic strategy (An et al., 2022). This is because the AMPK phosphorylation regulation mechanism allows the drug effect to take effect rapidly and activate the NRF2 antioxidant gene expression. Nutrient-responsive activation of AMPK and NRF2 influences fatty acid oxidation, glucose utilization, and cellular stress resistance—processes that are increasingly recognized as druggable in cardiovascular pharmacology (Qiu et al., 2023). Omics-based analyses indicate that nutrition-associated alterations in mitochondrial metabolites and redox-related enzymes correlate with disease severity and cardiovascular outcomes, reinforcing the translational relevance of these pathways (Regan et al., 2023).

Collectively, oxidative and mitochondrial signaling networks exemplify how nutritional inputs shape core pharmacological targets through regulation of cellular metabolism and stress responses.

## 3 AI-driven identification of nutrition-responsive biomarkers and drug targets

The increasing availability of large-scale molecular and clinical datasets has created unprecedented opportunities to elucidate nutrition-modulated mechanisms in cardiovascular disease (CVD) (Maiorino and Loscalzo, 2023; Sopic et al., 2023). However, the high dimensionality, heterogeneity, and non-linear structure of these data substantially limit the effectiveness of conventional statistical approaches (Drouard et al., 2024). Since mere pathway knowledge is insufficient to achieve the selection of biomarkers/targets, artificial intelligence (AI), particularly machine learning and network-based modeling, has become a key enabling technology for converting complex nutritional and molecular information into actionable biomarkers and therapeutic targets (Sabry et al., 2025).

### 3.1 Machine learning for biomarker prioritization

Machine learning (ML) methods provide powerful tools for identifying nutrition-responsive biomarkers by capturing complex, non-linear relationships among molecular features, dietary exposures, and cardiovascular outcomes (Louca et al., 2022; Navratilova et al., 2024). Supervised learning algorithms, such as random forests and gradient boosting models, have been widely applied to metabolomic and lipidomic datasets to prioritize features associated with cardiovascular risk and disease progression (Drouard et al., 2024; Moskaleva et al., 2022). Compared with traditional regression-based methods, machine learning models can enhance prediction performance through the input of dietary exposure, metabolites, clinical phenotypes, etc., and achieve more robust risk stratification by simultaneously integrating multiple molecular signals related to nutrition (Louca et al., 2022; Moskaleva et al., 2022).

Importantly, recent studies emphasize that ML-driven biomarker discovery is most effective when combined with biological interpretability rather than treated as a purely predictive exercise (Baron et al., 2025; Tarabanis et al., 2023). Feature importance analysis and model-agnostic interpretation techniques facilitate the identification of metabolites, lipid species, or inflammatory mediators that are not only statistically informative but also mechanistically linked to druggable pathways. Such approaches shift biomarker discovery from correlation-based screening toward hypothesis-generating frameworks with pharmacological relevance.

By prioritizing biomarkers that reflect nutrition-modulated molecular processes, ML approaches provide a foundation for identifying patient subgroups most likely to benefit from targeted pharmacological or combined nutritional–pharmacological interventions.

### 3.2 Network pharmacology and systems-level modeling

While single biomarkers offer diagnostic or prognostic value, cardiovascular pharmacology increasingly recognizes that disease progression is driven by coordinated perturbations across molecular networks (Reitz et al., 2023; Wang et al., 2023). Network pharmacology and systems biology approaches integrate nutrition-related molecular data with protein–protein interaction maps, signaling pathways, and drug–target databases to identify key regulatory nodes that serve as potential therapeutic targets (Wang et al., 2023; Nasirian and Menichetti, 2023).

In this context, AI-enabled network modeling allows the identification of hub genes, enzymes, or signaling complexes that mediate the effects of nutritional inputs on cardiovascular pathology. Rather than focusing on isolated molecular changes, these models highlight clusters of interconnected targets that may be more amenable to pharmacological modulation. This systems-level perspective aligns with the multi-target nature of cardiovascular therapies and supports rational combination strategies that integrate dietary modulation with drug intervention. Therefore, the multi-target strategy for complex networks naturally leads to a “combination and synergy” treatment framework. Through the interaction and mechanism complementarity among various targets, the overall therapeutic effect exceeds the sum of individual effects, achieving enhanced treatment efficacy.

Recent advances demonstrate that network-based approaches can reveal previously unrecognized links between nutrient-derived metabolites and established drug targets, offering opportunities for drug repositioning and target refinement within cardiovascular disease networks (Jin et al., 2025). These findings reinforce the value of AI-driven network pharmacology in bridging nutrition research and therapeutic development.

### 3.3 Multi-omics integration as a drug discovery accelerator

The integration of transcriptomic, proteomic, and metabolomic data represents a major frontier in cardiovascular drug discovery (Sopic et al., 2023; Zhan et al., 2023). Nutrition influences multiple molecular layers simultaneously, necessitating analytical frameworks capable of capturing cross-omic interactions (Navratilova et al., 2024; Kourak et al., 2024). AI-driven multi-omics integration methods, including matrix factorization and deep learning architectures, enable the identification of latent molecular patterns that link nutritional exposures to cardiovascular phenotypes (Maiorino and Loscalzo, 2023; Sabry et al., 2025).

By integrating diverse data types, AI approaches can uncover nutrition-responsive molecular signatures that converge on druggable enzymes, receptors, or signaling pathways (Sabry et al., 2025; Wang et al., 2023). Such integrative analyses reduce noise inherent in single-omics studies and enhance biological interpretability, facilitating the translation of nutritional insights into pharmacological hypotheses (Maiorino and Loscalzo, 2023; Drouard et al., 2024). Moreover, multi-omics integration supports the identification of causal pathways and regulatory hierarchies that are critical for target validation and therapeutic prioritization (Sabry et al., 2025; Wang et al., 2023).

Collectively, AI-driven ML, network pharmacology, and multi-omics integration form a complementary toolkit for discovering nutrition-responsive biomarkers and drug targets. Rather than serving as isolated analytical strategies, these approaches converge to enable a systematic, mechanism-oriented framework for cardiovascular pharmacology that leverages nutritional modulation as a source of therapeutic innovation.

## 4 Translational implications for cardiovascular pharmacology

Combining the insights of nutritional responsive molecules with AI-driven analysis is of great significance in cardiovascular pharmacology, especially in aspects such as precise prevention, treatment stratification, and target optimization. Target optimization encompasses concepts such as dosage, combination, and stratification of applicable populations (Reijnders et al., 2023). By reframing nutrition as a modulator of druggable pathways rather than a lifestyle variable, this framework bridges molecular nutrition research and translational pharmacology.

First, nutrition-modulated biomarkers offer opportunities to refine cardiovascular risk stratification and guide therapeutic decision-making (Xie et al., 2025). Traditional clinical risk models rely heavily on demographic and biochemical indicators that incompletely capture underlying molecular heterogeneity (SCORE2, 2021; Crea, 2021). In contrast, AI-prioritized biomarker panels derived from metabolomic and lipidomic profiles can reflect nutrition-sensitive biological states linked to lipid handling, inflammation, and mitochondrial metabolism (Wu et al., 2024; Gibson et al., 2025). Such biomarkers may identify patient subgroups with distinct pathway activation patterns, thereby informing the selection or intensification of lipid-lowering, anti-inflammatory, or metabolic therapies (Reijnders et al., 2023; Xie et al., 2025). Emerging research evidence suggests that molecular typing may be superior to traditional risk scores in predicting treatment response, supporting the value of nutrient responsiveness biomarkers for clinical use in cardiovascular drug therapy.

Second, understanding how nutritional inputs modulate established drug targets provides a mechanistic basis for synergistic intervention strategies (Reijnders et al., 2023). Many core cardiovascular drug targets—including PCSK9, PPARs, AMPK, and inflammatory signaling nodes—are sensitive to nutrient availability and metabolic context (Németh et al., 2023; Fuior et al., 2023; Rakoubian et al., 2025). AI-enabled integration of nutritional and pharmacological data can elucidate how dietary modulation influences target expression, pathway crosstalk, and drug efficacy (Di Martino and Delmastro, 2023). This insight is particularly relevant for optimizing combination strategies, in which pharmacological agents are complemented by targeted nutritional modulation to enhance therapeutic benefit or reduce adverse effects (Reijnders et al., 2023; Xie et al., 2025). Rather than advocating generalized dietary recommendations, this approach emphasizes pathway-specific modulation aligned with pharmacological mechanisms.

Third, The AI-driven framework can prioritize the screening of drug targets related to nutrition. By integrating metabolites, gene expression and drug-target data through network analysis, it identifies regulatory hubs that possess both biological centrality and drug feasibility. This way, it provides candidate targets for new drug development or the reutilization of existing drugs in the nutritional regulation pathways. This systematic approach aligns with the multi-target regulatory trend in modern drug discovery for complex diseases such as atherosclerosis.

From a broader translational perspective, AI-enabled nutritional pharmacology supports a shift toward precision cardiovascular medicine (Reijnders et al., 2023). By linking molecular nutrition to druggable mechanisms, this framework moves beyond population-level dietary advice and toward individualized intervention strategies grounded in molecular phenotyping. This paradigm is particularly relevant in addressing residual cardiovascular risk and inter-individual variability in drug response, two persistent challenges in clinical cardiology.

Nevertheless, translation into clinical practice requires careful consideration of methodological and regulatory constraints (Cinà et al., 2025). Biomarkers and targets identified through AI-driven analyses must undergo rigorous validation in experimental models and clinical cohorts to establish causality and reproducibility. Moreover, integration into clinical workflows will depend on assay standardization, interpretability of AI models, and demonstration of incremental clinical value over existing approaches. In other words, it involves systematically comparing the new model with the existing authoritative tools in the same population. Through multiple dimensions such as discrimination, calibration, and clinical utility, its performance advantages are verified. Eventually, it is proven that these advantages can be translated into more accurate clinical decision support.

In summary, the convergence of nutrition-responsive molecular profiling and AI-driven discovery provides a translationally relevant framework for cardiovascular pharmacology. By enabling biomarker-guided stratification, informing target modulation, and accelerating drug discovery, this approach has the potential to reshape therapeutic strategies for cardiovascular disease while maintaining a clear mechanistic and pharmacological focus.

## 5 Challenges and future perspectives

Despite the growing promise of integrating nutrition-responsive molecular profiling with artificial intelligence (AI)–driven discovery, several challenges must be addressed before this framework can be fully translated into cardiovascular pharmacology (Ruxton, 2022; Mirmiran et al., 2021). A primary limitation lies in the predominance of associative findings (Feng, 2023). Most existing studies linking nutritional factors to cardiovascular biomarkers or targets rely on cross-sectional or observational designs, which restrict causal inference. While AI methods excel at pattern recognition and feature prioritization, they do not inherently distinguish causation from correlation (Shi and Norgeot, 2022; Sanchez et al., 2022). Bridging this gap will require closer integration of AI-guided discovery with experimental validation, including mechanistic studies in cellular and animal models and well-designed interventional trials (Stadelmaier et al., 2022; Hernán et al., 2022).

Data heterogeneity represents another major obstacle (Yamamoto et al., 2023; Alseekh et al., 2021). Nutritional exposures, omics platforms, and clinical phenotyping vary widely across cohorts, complicating model generalizability and reproducibility. Differences in dietary assessment methods, population structure, and analytical pipelines can introduce substantial bias, limiting cross-study comparability (Liu et al., 2020),For instance, different dietary assessment tools are suitable for different research purposes, but the results may vary significantly. Future efforts should emphasize standardized data collection, harmonized preprocessing workflows, and transparent reporting to improve robustness and facilitate external validation (Collins et al., 2024). Advances in federated learning and transfer learning may help mitigate data fragmentation while preserving cohort-specific information (Oh and Nadkarni, 2023; Sharma et al., 2024).

Model interpretability is a further critical concern for clinical translation. Although complex machine learning and deep learning architectures can capture high-dimensional interactions, their “black-box” nature hampers biological insight and regulatory acceptance (Amann et al., 2022). In the context of pharmacology, interpretability is essential not only for clinician trust but also for target validation and drug development. The increasing adoption of interpretable AI approaches opens up a promising path for the field to more clearly link nutritional regulatory inputs, molecular pathways, and therapeutic targets.

Looking forward, the integration of longitudinal multi-omics data with dynamic nutritional profiling represents an important frontier (Babu and Snyder, 2023; Mohr et al., 2024). Temporal analyses can capture pathway adaptation, treatment response, and disease progression more accurately than static snapshots. Coupled with AI-driven causal inference frameworks, such approaches may clarify how nutritional modulation interacts with pharmacological interventions over time. Additionally, incorporating real-world data and digital health metrics may further enhance the clinical relevance of AI-enabled nutritional pharmacology (Liu and Panagiotakos, 2022; Simon et al., 2022).

In conclusion, while significant challenges remain, the convergence of nutrition, AI, and pharmacology offers a compelling opportunity to advance precision cardiovascular therapeutics. By reframing nutrition as a source of modifiable molecular signals and embedding AI within mechanistic discovery pipelines, future research can move beyond descriptive associations toward actionable biomarkers and druggable targets. Addressing current methodological and translational barriers will be essential for realizing the full potential of this emerging paradigm in cardiovascular disease management.
